# Potassium and Boron Co-Doping of g-C_3_N_4_ Tuned CO_2_ Reduction Mechanism for Enhanced Photocatalytic Performance: A First-Principles Investigation

**DOI:** 10.3390/molecules29225339

**Published:** 2024-11-13

**Authors:** Gang Fu, Wenqing Zhen, Hongyi Wang, Xin Zhou, Li Yang, Jiaxu Zhang

**Affiliations:** 1 Xinjiang Key Laboratory of Clean Conversion and High Value Utilization of Biomass Resources, School of Chemistry and Chemical Engineering, Yili Normal University, Yining 835000, China; 20b925088@stu.hit.edu.cn; 2 State Key Laboratory of Urban Water Resource and Environment, MIIT Key Laboratory of Critical Materials Technology for New Energy Conversion and Storage, School of Chemistry and Chemical Engineering, Harbin Institute of Technology, Harbin 150001, China; 20b925102@stu.hit.edu.cn (W.Z.); wanghongyi999123@163.com (H.W.); zhoux@hit.edu.cn (X.Z.)

**Keywords:** g-C_3_N_4_, CO_2_PR, metallic–nonmetallic co-doping, reaction mechanism

## Abstract

Graphitic phase carbon nitride (g-C_3_N_4_, abbreviated as CN) can be used as a photocatalyst to reduce the concentration of atmospheric carbon dioxide. However, there is still potential for improvement in the small band gap and carrier migration properties of intrinsic materials. K-B co-doped CN (KBCN) was investigated as a promising photocatalyst for carbon dioxide reduction via the Density Functional Theory (DFT) method. The electronic and optical properties of CN and KBCN indicate that doping K and B can improve the catalytic performance of CN by promoting charge migration and separation. In terms of the Gibbs free energy change, the CO_2_ reduction reaction catalysed by KBCN results in CH_3_OH, and its optimal pathway is CO_2_ → *CO_2_ → *COOH → CO → *OCH → HCHO → *OCH_3_ → CH_3_OH. Compared with CN, the doping elements K and B shift the rate-determining step from CO_2_ → *CO_2_ to *CO_2_ → *COOH. The K and B elements co-doping tuned the charge distribution between the catalyst and the adsorbate and reduced the Gibbs free energy of the rate-determining step from 1.571 to 0.861 eV, suggesting that the CO_2_ reduction activity of KBCN is superior to that of CN. Our work provides useful insights for the design of metallic–nonmetallic co-doped CN for photocatalytic CO_2_ reduction (CO_2_PR) reactions.

## 1. Introduction

With the increasing trend of industrialisation and urbanisation, the concentration of CO_2_ in the atmosphere is rising [1]. This has led to a number of environmental problems, such as the greenhouse effect, melting glaciers, global climate extremes, and acidification of ocean waters [2,3,4,5]. At the same time, the burning of non-renewable resources, such as coal and oil, has led to an ongoing energy crisis along with the excessive emission of CO_2_ into the atmosphere [6]. To solve these problems, carbon dioxide must be captured and converted into useful industrial commodities to establish a carbon cycle and achieve carbon neutrality [7,8]. Reducing carbon dioxide emissions has, therefore, become a new research hotspot and challenge, with many researchers now using a range of measures such as thermocatalysis [9], photocatalysis [10,11,12,13,14], electrocatalysis [15], biocatalysis [16], and organocatalysis [17] to convert CO_2_ into value-added products and fuels. Of these, photocatalysis has attracted worldwide attention for its green, environmentally friendly, and sustainable benefits.

Two-dimensional (2D) materials such as metal oxides [18], layered double hydroxide [13,19], carbon nitrides [20,21,22,23,24,25], and graphene-based compounds [26] can be used as potential materials for photocatalytic processes due to their high chemical and thermal stability and high specific surface area. Among these, graphitic phase carbon nitride (g-C_3_N_4_, abbreviated as CN) has superior thermochemical stability and photocatalytic activity due to the strong covalent interactions between the carbon and nitrogen elements and the π-conjugated structure [27,28,29]. However, the disadvantages of a large band gap and the easy recombination of electrons and holes limit its application. To overcome this problem, surface modification methods such as defect engineering [21,22], elemental doping [30,31,32,33,34,35,36], surface loading [37,38], and heterostructure building [39] have been proposed. Among them, heteroatom doping has been widely investigated as a well-known strategy to improve the photocatalytic performance of materials. Praserthdam et al. [40] investigated the photocatalytic CO_2_ reduction reaction of CN singly doped with nonmetallic B/O/P/S elements and found that nonmetallic doping was effective in suppressing carrier complexation and, thus, improving the methane yield. Ohno et al. [34] prepared B-doped carbon nitride compounds and found the same regularity, with boron enhancing the reduction in CO_2_ to ethanol by improving electron–hole separation. However, both studies showed that nonmetallic elements alone did not significantly improve the band gap value. Gan and Xu’s group [41,42] investigated the bi- and tri-nonmetallic co-doping of CN and found that multi-element doping can establish an efficient charge transfer channel through a synergistic effect and significantly reduce the band gap. The variety of metallic elements is much richer than that of nonmetallic elements, as can be observed from the periodic table. Therefore, CN doped with metallic elements has also been extensively studied. Yu et al. [43] prepared Cu-doped CN and found that copper could form polycentric bonds with the surrounding nitrogen and carbon, using copper’s ability to change valence state to facilitate the rapid reduction in CO_2_ to CO. Mi’s group [44] studied alkali metal K-doped CN and found that potassium doping increased the crystallinity of the crystals, thus improving the photocatalytic ability. In addition to metal mono-doping, Li et al. [31] investigated Ni-Cu and Ni-Mn co-doped CN catalysts, and the results showed that the synergistic effect of the bimetals can modulate the structure of the intermediate adsorption state and improve the yield of CO while inhibiting the ability of hydrogen evolution. Nonmetal or metal co-doping studies have been extensively reported and have confirmed that elemental double doping provides a greater performance enhancement than elemental single doping. However, very few studies have been carried out on nonmetallic and metallic elemental co-doping systems, such as the P-Na [45] co-doping and the K-B [25] co-doping CN. The former has been applied to the hydrogen evolution reaction, while the latter has been applied to the production of cyclic carbonate. In addition, the application of metallic–nonmetallic co-doped CN in the multi-reaction pathway reduction process of CO_2_ is rarely studied, and the mechanism is unclear and needs further exploration.

In this work, K and B co-doped CN (abbreviated as KBCN) was chosen as a catalyst to study the reduction process of CO_2_ based on the properties of the elements. We investigate the possible reaction pathways for the reduction in CO_2_ to CH_3_OH by CN/KBCN catalysts using Density Functional Theory (DFT). The Gibbs free energy change (Δ*G*) was calculated to understand the best reaction pathway for the reduction in CO_2_ to CH_3_OH. In addition, the effects of doping K and B elements on the structure, electronic properties, and photocatalytic CO_2_ reduction (abbreviated as CO_2_PR) activity are investigated and discussed.

## 2. Results and Discussions

### 2.1. Geometric Structure and Thermodynamic Stability

**Figure 1:**
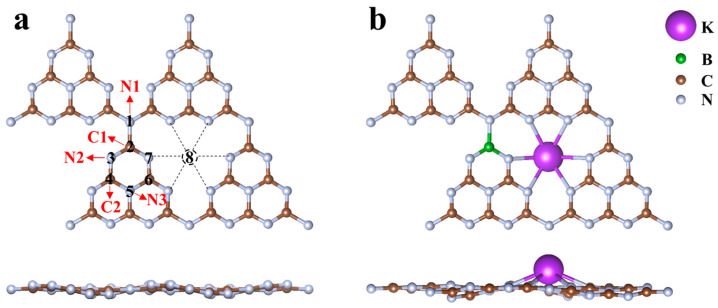
(**a**) Top and side views of geometric structure of CN and the possible doping sites [1(N1), 2(C1), 3(N2), 4(C2), 5(N3), and 8(interstitial)]. (**b**) Top and side views of the most stable KBCN geometric structure, where B atoms replace C1 and K atoms dope into the interstitial sites.

The experimental results of Wang et al. [25] showed that CN doping with one K and one B atom showed the best performance in catalysing the CO_2_ process. Therefore, we designed a catalyst with a similar concentration to that in Wang et al., and its chemical formula is KBC_23_N_32_. The optimised structures of CN and KBCN are shown in Figure 1. In previous experiments, the K element was doped into the CN in an interstitial position [44], while the B element was always doped into the CN in an alternative C position [34]. In this work, the substitution doping model of CN was constructed for five different B-doping sites (1, 2, 3, 4, and 5 representing N1, C1, N2, C2, and N3, respectively), with K atoms doped into the interstitial positions (site 8) because of their atomic size. The N1 atom is connected to the three nearest heptazine rings, N2 is two-fold-coordinated to the two C atoms of the C_6_N_7_- unit, the N3 atom is three-fold-coordinated to the three C atoms of the C_6_N_7_- unit, and C1 and C2 are three-fold-coordinated to the three N atoms of the C_6_N_7_- unit. To evaluate the possible doping sites, the formation energy (*E*_form_) of the doping system KBCN is calculated according to the calculation method of Equation (1) in Section 3.

**Figure 2:**
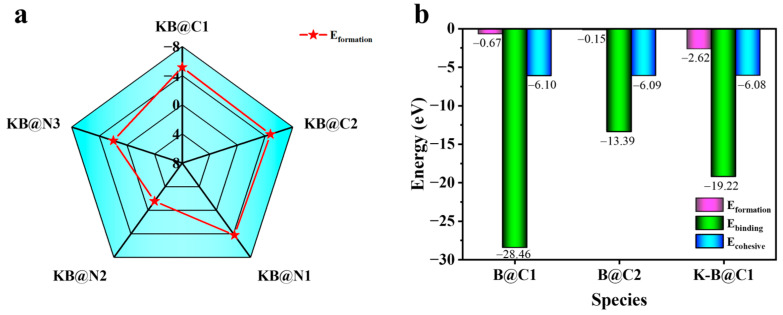
(**a**) Formation energy of K and B atoms co-doped at different sites of CN. (**b**) Formation (pink), binding (green), and cohesion (blue) energies of B@C1, B@C2, and K-B@C1 catalysts.

On the one hand, elements K and B can be doped into CN simultaneously, and the *E*_form_ values for the KB@C1-, KB@C2-, KB@N1-, KB@N2-, and KB@N3-doped systems were −5.15, −4.77, −4.23, 1.53, and −1.96 eV, respectively, as shown in Figure 2a. The formation of the C-site doped system was, thus, more stable than that of the N-site doped system, which is in good agreement with theoretical and experimental studies [25]. On the other hand, to further investigate the ease of introducing K atoms after doping with B atoms, we first calculated the formation, binding, and cohesion energies of B atoms, replacing C atoms at different positions to screen out the most stable B@C catalysts to introduce potassium atoms. The results in Figure 2b show that the C1 position of doped B (B@C1) has a formation, binding, and cohesion energy of −0.67, −28.46, and −6.10 eV, respectively, values which are lower than the C2 position of doped B (B@C2), e.g., −0.15, −13.39, and −6.09 eV. Thus, B preferentially replaces the C1 position, which is consistent with previous studies [46]. Subsequently, we calculated the three energies of K doping into B@C1 catalysts to investigate the effect of the first doping element on the second doping element. The formation, binding, and cohesion energy calculated for the introduction of the K atom into B@C1 (K-B@C1) was −2.62, −19.22, and −6.08 eV, respectively, as shown in Figure 2b. The fact that all three energies are less than zero means that the K atom is still easily doped into the system after the B atom has been doped with CN. In addition, previous investigators [35] found that elementally doped monolayer CN or bilayer CN had a negligible effect on the adsorption energy of the key step *COOH, where the * symbol indicates that the intermediates can be adsorbed on CN, so monomolecular layers CN and KBCN were also chosen to study the specific process of CO_2_ reduction in this study.

To explain how the doping elements affect the properties before and after doping, the optimised bond length parameters for CN and KBCN are shown in Table S1 and compared with the experimental [33] and theoretical [21,44] values. Evidently, the optimised geometrical parameters for pure CN are in good agreement with the results in the literature. The maximum difference between the cell parameter (7.35 Å) and the theoretical (7.13 Å) and experimental (7.30 Å) values is 0.22 and 0.05 Å, i.e., a relative deviation of 3% and 0.7%, respectively. The largest difference in bond length is in the N2-C2 bond (1.358 Å), with a maximum difference of 0.028 Å from the theoretical value (1.330 Å), a relative deviation of 2%. The deviations in geometry are within acceptable limits, further validating the reliability of the calculation method and parameter settings. Table S1 shows the change in key bond lengths from CN to KBCN. The elemental introduction changes the local geometry of the doping site compared to pure CN, with little effect on the other sites. The dramatic change lies in the B-N bond, where N1-B (1.676 Å), B-N2 (1.445 Å), and B-N (1.442 Å) are longer than N1-C1 (1.477 Å), C1-N2 (1.350 Å), and C1-N (1.349 Å) at the same positions, due to the fact that the B atom (r_a_ = 0.88 Å) has a larger geometrical radius than the C atom (r_a_ = 0.77 Å). In addition, B is bonded to the three surrounding N atoms in the form of sp^2^ hybridisation, leaving an empty 2p orbital that can be used as a carrier trapping centre and also as an adsorption site for gas molecules. The introduction of K results in a larger cavity, with a longer bond length for N-K (2.673 Å) than for N-interstitial (2.522 Å), which is attributed to the local geometric distortion of CN caused by the doping of K. In general, as the degree of geometrical distortion increases, the repulsion between the lone pairs of electrons of the cavity N atoms decreases, and the material becomes more stable. Finally, the variation in energy with time for CN and KBCN at room temperature and 500 K was simulated using ab initio molecular dynamics (AIMD), as shown in Figure S1 and Animations S1–S4. The energy was found to fluctuate within 1 eV over a range of 5000 fs, and the geometrical configurations were similar before and after the simulations, indicating good thermodynamic stability.

### 2.2. Electronic Properties of CN and KBCN

**Figure 3:**
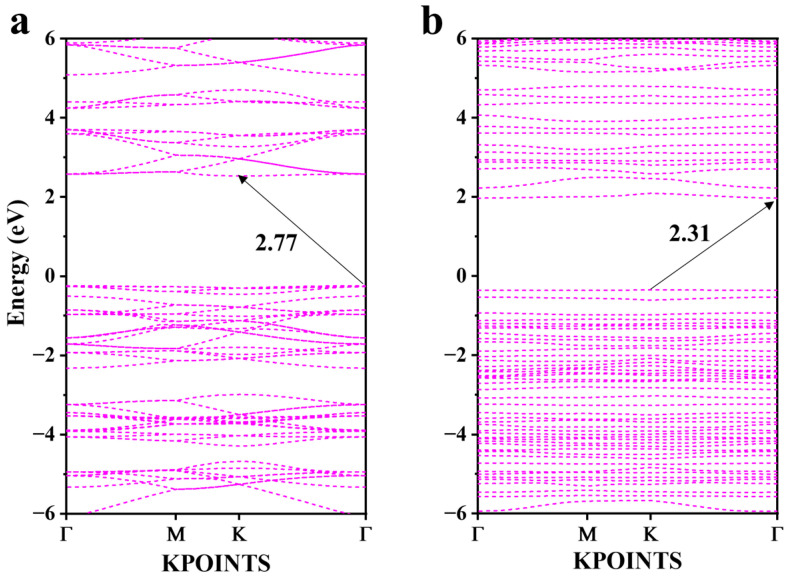
Band structure of (**a**) CN and (**b**) KBCN.

**Figure 4:**
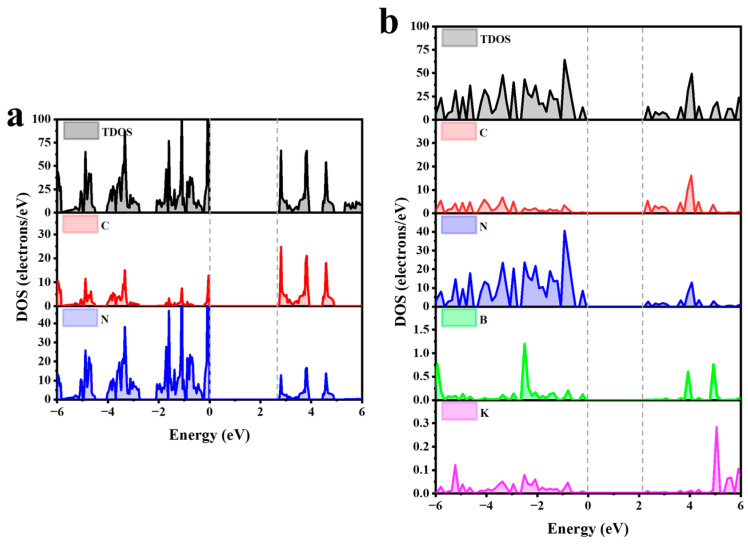
Local density of states of (**a**) CN and (**b**) KBCN.

The energy band structures and density of states of CN and KBCN were calculated via the HSE06 method and are shown in Figure 3, Figure 4 and Figure S2. It is worth noting that the band gap of CN (2.77 eV) calculated using the HSE06 function is similar to the experimental data (2.79 eV) [47]. The band gap of KBCN (2.31 eV) is reduced by co-doped K and B atoms compared to CN, as shown in Figure 3. Additionally, the band gap of KBCN shows also improvement over singly B-doped CN (2.63 eV) [34], and K-doped CN (2.72 eV) [47].

A further analysis of the density of states for CN shows that VB is dominated by N atoms and is contributed by N_2px_ and N_2py_ orbitals in N. CB is mainly contributed by C_2pz_ orbitals in C atoms, with a small amount of CB being from N atoms as shown in Figure 4a and Figure S2a,b. For KBCN, VB is composed of N and B atoms, and CB is composed of C and N atoms, as depicted in Figure 4b and Figure S2e,f. Observing the DOS of KBCN, the electronic push–pull of the B, C, and N elements drives the conduction band downward shift resulting in a reduced band gap. This broadens the range of visible light utilisation and, thus, improves the CO_2_ reduction process.

**Figure 5:**
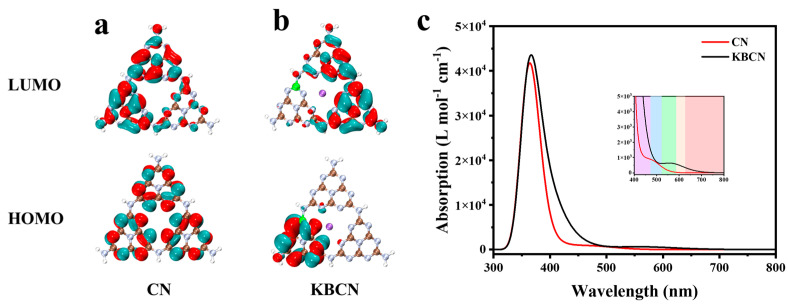
LUMO and HOMO of (**a**) CN and (**b**) KBCN. Atomic color codes B, C, N, K are represented by green, brown, silver, and purple, respectively. (**c**) Optical absorption behaviors of CN and KBCN.

Figure 5a,b show the highest occupied molecular orbital (HOMO) and the lowest unoccupied molecular orbital (LUMO) of CN and KBCN. As shown in Figure 5a, HOMO is mainly composed of the p orbitals of N atoms, while LUMO is mainly composed of the p orbitals of C atoms, with little contribution from N atoms. Occupied and vacant orbitals are independently distributed in each heptazine structural unit, which is consistent with the local density of the state. In other words, under light irradiation, light-generated electrons are not transferred from one heptazine (C_6_N_7_) unit to an adjacent unit via a bridging N atom. This means that the excited electron–hole pair of each heptazine unit is easily compounded, so the photocatalytic efficiency of g-C_3_N_4_ is weak. For KBCN, the HOMO is predominantly located on the B-doped heptazine unit, as shown in Figure 5b. In contrast, LUMO is mainly localised on the two heptazine units that do not contain B elements. These results are consistent with the LDOS analysis that the B atom is involved in VB. In summary, the orbital distribution of CN was significantly separated after doping with B and K, resulting in a higher ability to separate photogenerated h^+^/e^−^ pairs and improved photocatalytic efficiency. In addition, the optical absorption curves of CN and KBCN are shown in Figure 5c, and the two catalysts have a similar optical absorption peak near 365 nm. However, the optical absorption curves of the KBCN catalyst decay more slowly compared to CN, indicating that the KBCN can extend the visible light response range. The local detail magnification in Figure 5c allows the analysis of the integrated area of the CN and KBCN catalyst light absorption curves at visible light (about 400 nm~780 nm). Compared with CN, the addition of K and B elements enhances the absorption strength of the catalyst in the visible region, indicating that metallic–nonmetallic-co-doped CN can enhance photocatalytic performance.

### 2.3. Band Edge Positions of CN and KBCN

**Figure 6:**
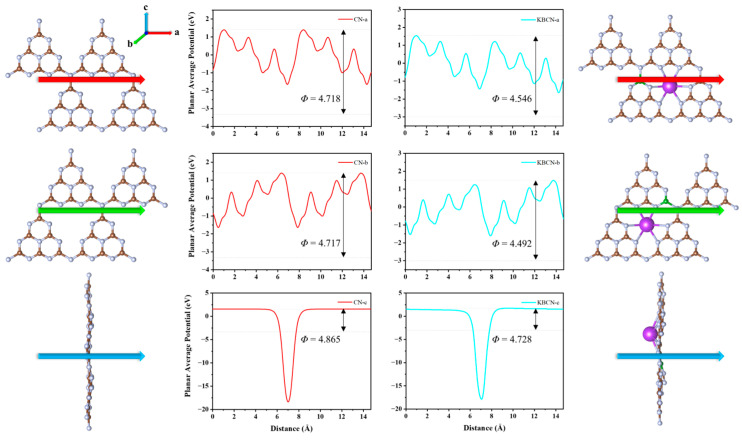
The planar average potential was calculated for the three directions of CN (**left**) and KBCN (**right**). The a and b directions are parallel to the plane, while the c directions are perpendicular to the plane. The colours for a, b, and c are red, green, and blue, respectively.

The transportability of photogenerated carriers in CN and KBCN was investigated by calculating the planar average potential through K and B elements doped into the 3s-triazine ring to obtain doping configurations. From CN (Figure 6, left) to KBCN (Figure 6, right), the work function changes from 4.718, 4.717, and 4.865 to 4.546, 4.492, and 4.728 eV in the three directions of a, b, and c, respectively. The decrease in the work function indicates that the KBCN photogenerated carrier transport performance is better than that of CN. This improved charge transport capability is conferred by the advantages of the KBCN crystal structure, i.e., a more ordered structural arrangement due to the doping of K and B elements. Furthermore, the decrease in the work function is higher in the a (0.172 eV) and b (0.225 eV) directions parallel to the structure plane than in the c (0.137 eV) direction perpendicular to the plane, as shown in Table S2, indicating that the charge transport is far more favourable in the a and b directions than in the c direction.

**Figure 7:**
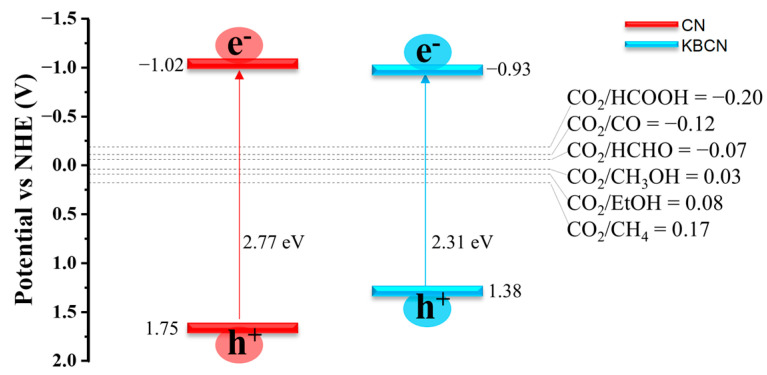
Calculation of the CBM and VBM band edge positions for CN using the HSE06 function and electrode potential for the reduction in CO_2_ to hydrocarbons.

Semiconductor materials used as photocatalysts for CO_2_ reduction need to have suitable band edge positions to match the CO_2_/hydrocarbon potentials. The VB and CB extreme (VBM and CBM) edge positions of CN and KBCN were calculated relative to the normal hydrogen electrode potential using Equations (4)–(7) in Section 3. As shown in Figure 7, the CBM of CN and KBCN is −1.02 and −0.93 V, respectively, values which are higher than the potential for CO_2_ reduction to various substances such as CO, HCOOH, HCHO, CH_3_OH, CH_4_, and EtOH. Meanwhile, the VBM of CN and KBCN is 1.75 and 1.38 V, values which are lower than the reduction potentials of CO_2_. Therefore, CN and KBCN can be used as potential photocatalysts for the reduction in CO_2_ to various hydrocarbons. Although the CBM of KBCN is slightly lower than that of CN, its potential is still higher than the reduction potential of CO_2_. It also has an enhanced range of visible light utilisation due to the reduced band gap. KBCN is, therefore, more suitable than CN for CO_2_ reduction because of its smaller band gap value, which is 2.77 eV for CN and only 2.31 eV for KBCN.

### 2.4. Catalytic Performance for the CO_2_PR on CN and KBCN

**Figure 8:**
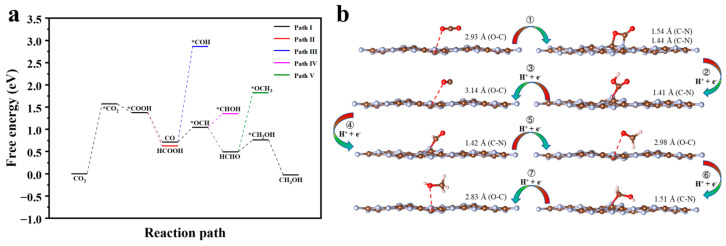
(**a**) Gibbs free energy diagrams of photocatalytic CO_2_ reduction in CN. (**b**) The representative geometries of the stable points in the specific reduction process for CO_2_ on CN. Note: The * symbol shows that the intermediate can be adsorbed on the CN.

Azofra and Zhang et al. [21,48] suggest that CN photocatalytic CO_2_ reduction follows a six-electron reaction pathway with the following scheme: CO_2_ → *COOH → CO → HCO* → HCHO → CH_3_O* → CH_3_OH. However, in the proton-coupled electron transfer (PCET) step, there are two types of coupling, one between hydrogen and carbon atoms and the other with oxygen atoms. The Gibbs free energy changes for different reduction reaction paths of CN-photocatalysed CO_2_ are, therefore, re-examined in this paper (see Figure 8a), and the geometrical structures of the adsorption states of the optimal and secondary paths are shown in Figure 8b and Figure S3a, respectively. In addition, the chemisorption state of CO_2_ on CN was calculated to explore how this step affects the CO_2_ reduction process. The results show a total of five reaction pathways. Path I, where CO_2_ is eventually reduced to CH_3_OH, has a value of ∆*G* = −0.026 eV, which is less than zero, meaning that the reaction is exergonic and the reduction product CH_3_OH is, therefore, thermodynamically stable. Path II is reduced to HCOOH through a two-electron reduction step; however, with a Δ*G* of 0.626 eV, the positive value for this step indicates that it is an endothermic process, making it a less favourable reaction pathway. The stabilisation points for Path III, Path IV, and Path V steps are *COH, *CHOH, and *OCH_3_, respectively, with ∆*G* of 2.864, 1.354, and 1.825 eV, respectively. However, compared to the stabilisation points of the same electron reduction step, namely *OCH (Δ*G* = 1.046 eV), HCHO (Δ*G* = 0.495 eV), and CH_2_OH (Δ*G* = 0.764 eV), the values are much higher, and these three pathways are secondary pathways. The three lower stability points mentioned earlier all belong to Path I, which is also the most favourable pathway for the reduction in CO_2_ by CN. To analyse Path I in the context of Figure 8b, firstly, we measure the CO_2_ changes from the physisorbed state to the chemisorbed state after step ①, which requires the absorption of 1.571 eV of energy and is the decisive step of the whole reaction process. The chemisorbed CO_2_ then couples with h^+^/e^−^ to give *COOH, i.e., step ②, which is an exergonic reaction and releases 0.196 eV of energy. *COOH continues to couple with h^+^/e^−^ to give CO + H_2_O, i.e., step ③, which releases 0.663 eV and is a thermodynamically spontaneous process. CO continues to couple with h^+^/e^−^, at which point the hydrogen proton has two couplings, one of which is with the oxygen atom to *COH (Path III) and the other with the carbon atom to *OCH (step ④). The latter has a lower energy, so it absorbs 0.334 eV of energy and is reduced to *OCH. *OCH remains coupled in both ways, and the energy preference is for coupling to the C atom, i.e., step ⑤ releases 0.551 eV of energy to give the HCHO product. The reduction in formaldehyde continues to give *CH_2_OH, i.e., step ⑥, where the proton is preferentially coupled at the O site and absorbs 0.269 eV of energy. This is probably due to the formation of hydrogen bonds between the H in the generated hydroxyl group and the catalyst. Eventually, *CH_2_OH couples to h^+^/e^−^ and undergoes a six-electron reduction step to CH_3_OH, i.e., step ⑦, which releases 0.79 eV energy to give the final thermodynamically stable product. Figure S4a shows that the CO_2_, CO, HCHO, and CH_3_OH stabilisation points barely exchange electrons with the catalyst and are in the physisorbed state. In contrast, *CO_2_, *OCH, and *CH_2_OH were adsorbed by the catalyst in a chemisorbed state with significant and sequentially increasing electron transfer. The thermodynamic curves further show that for the formation of the stabilisation point in the physisorbed state, energy is released, and conversely, the stabilisation point in the chemisorbed state requires energy absorption.

**Figure 9:**
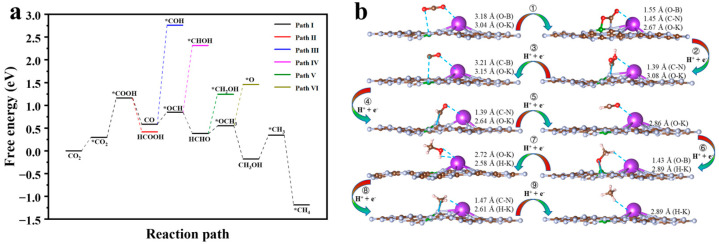
(**a**) Gibbs free energy diagrams of photocatalytic CO_2_ reduction in KBCN. (**b**) The representative geometries of the stable points in the specific reduction process for CO_2_ on KBCN. Note: The * symbol shows that the intermediate can be adsorbed on the KBCN.

Wang et al. synthesised KBCN experimentally [25], and this study explores the advantages of KBCN for photocatalytic CO_2_ reduction from a theoretical perspective. The complete eight-electron process of the reduction in CO_2_ by KBCN is considered. Figure 9a,b and Figures S3b and S4b show the Gibbs free energy changes and the geometric and electronic structure changes in the reduction process. The calculations show that Path II, Path III, Path IV, Path V, and Path VI are the secondary reaction paths. Path II undergoes a two-electron reduction step to reduce CO_2_ to HCOOH with ∆*G* = 0.415 eV. It is, therefore, a heat-absorbing reaction and a thermodynamically non-spontaneous process. The intermediates undergoing Path III, Path IV, Path V, and Path VI are *COH, *CHOH, *CH_2_OH, and *O, respectively, corresponding to Gibbs free energies of 2.761, 2.315, 1.242, and 1.460 eV. However, the ∆*G* of Path I is lower than that of Path III–VI for the same electron reduction steps, where the ∆*G* of *OCH, HCHO, *OCH_3_, and CH_3_OH steps are 0.850, 0.382, 0.552, and −0.178 eV, respectively. Therefore, the CO_2_ reduction process mainly follows Path I. The CO_2_ is first adsorbed onto the KBCN in the physisorbed state, where the O atoms in the CO_2_ are mainly attracted to the B and K atoms in the catalyst. After step ①, the physisorbed CO_2_ changes to chemisorption, where the O and C in the CO_2_ are captured by the B and N in the KBCN, a process that absorbs 0.3 eV of energy. This is followed by step ②, which absorbs 0.861 eV of energy to produce *COOH, the decisive step in the reduction in CO_2_ by KBCN. In step ③, *COOH continues to couple h^+^/e^−^ to obtain CO + H_2_O, which releases 0.574 eV of energy and is a thermodynamically spontaneous process. The coupling of h^+^/e^−^ then diversifies, with coupling to the O atom of the catalyst giving *COH (Path III) and coupling to the C atom giving *OCH. The optimal path forms *OCH (step ④), absorbing 0.263 eV energy. In step ⑤, h^+^/e^−^ is still coupled to the energetically advantageous C site rather than the O site and undergoes a four-electron reduction process to give HCHO, which releases 0.468 eV of energy. Step ⑥ is similar to the previous step, with h^+^/e^−^ coupling at the C site to give *OCH_3_ and absorb 0.17 eV of energy. The stabilisation point of this process is different from that of CN because the hydroxyl H in *CH_2_OH in KBCN is further away from the catalyst, weakening the hydrogen bonding interaction. In addition, the O atom in KBCN interacts more strongly with the B atom, resulting in a lower energy of *OCH_3_. In step ⑦, h^+^/e^−^ combines with oxygen atoms to form CH_3_OH products rather than C atoms to form *O + CH_4_. This process releases 0.730 eV of energy and produces a thermodynamically stable CH_3_OH, ∆*G* = −0.178 eV. To investigate the complete eight-electron reduction in CO_2_, CH_3_OH continues to couple with h^+^/e^−^ in step ⑧ to give *CH_3_, which absorbs 0.522 eV of energy and is thermodynamically non-spontaneous. Finally, *CH_3_ is reduced to CH_4_ (step ⑨) by h^+^/e^−^ while releasing 1.462 eV of energy. Although ∆*G* = −1.118 eV for CH_4_, the KBCN is also eventually reduced to CH_3_OH because step ⑧ is a heat-absorbing process. The calculations show that for KBCN, h^+^/e^−^ prefers to couple with the C atom. The thermodynamic curves show that the formations of *COOH, *OCH, *OCH_3_, and *CH_3_ intermediates are heat-absorbing processes, whereas the formations of CO, HCHO, CH_3_OH, and CH_4_ products are exergonic processes.

**Figure 10:**
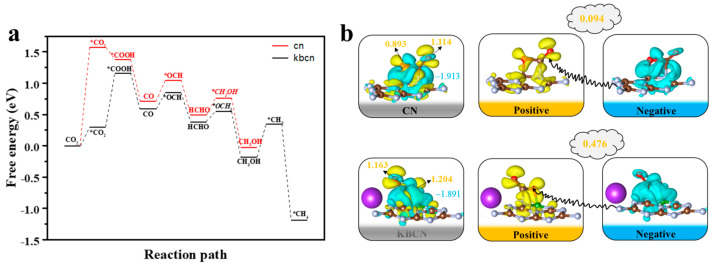
(**a**) Gibbs free energy diagram of the optimal reaction path for photocatalytic CO_2_ reduction by CN and KBCN. (**b**) Charge difference density (CDD) diagram for CO_2_ adsorption on CN and KBCN, where yellow/cyan indicates electron gain/loss, and the quantitative charge transfer number is based on the Bader charge. Note: The * symbol shows that the intermediate can be adsorbed on the CN and KBCN.

To verify the effect of B and K doping, the CO_2_PR pathways of CN and KBCN were compared and are shown in Figure 10a. Evidently, the rate-determining step catalysed by CN is CO_2_ → *CO_2_, with a Δ*G* of 1.571 eV. B and K doping changed the rate-determining step to *CO_2_ → *COOH and the Δ*G* of the rate-determining step decreased to 0.861 eV, indicating that the B- and K-doped CN-catalysed CO_2_PR is more favourable than CN. To investigate the reasons for this, we performed charge difference density calculations for the key stability point *CO_2_. Figure 10b shows that the KBCN catalyst can transfer 0.476 e to CO_2_, while the CN catalyst can only transfer 0.094 e to CO_2_, with the former transferring about five times as many electrons as the latter. This changes the rate-determining step and lowers the Gibbs free energy, thereby promoting the CO_2_ reduction process. Therefore, the co-doping of K and B elements can enhance the photocatalytic activity of CN. In addition, since the CO_2_PR reaction and hydrogen evolution reaction (HER) are in competition, we also calculated the free energy profiles of the HER for CN and KBCN. The results in Figure S5 show that the rate-determining steps of the HER process for CN and KBCN are 1.802 and 1.993 eV, respectively. The KBCN catalyst inhibits the HER process while promoting the CO_2_PR reaction compared to CN.

## 3. Computational Method

Density Functional Theory (DFT) calculations provide an efficient tool for investigating catalytic reaction pathways by examining individual primitive reaction steps. The mechanism of CN/KBCN on CO_2_PR was studied via DFT methods, which were implemented in the Vienna Ab initio Simulation Package (VASP) code [49]. The primitive cell of the CN catalyst was taken from the materials project database, while the supercell (2 × 2 × 1) was a periodic repetition of the primitive cell in the x and y directions with a 15 Å vacuum layer added in the z direction to avoid spurious interactions between neighbouring crystal lattice cells. A generalised gradient approximation (GGA) [50] with a Perdew–Burke–Ernzerhof (PBE) function [51] was used for all geometric optimisations. A previous study described the interaction between the effective inner core electron and the valence electron using the projected plus plane wave (PAW) pseudopotential [52]. The PBE + D3 method [53] with the Grimme van der Waals correction was employed because of the weak interactions between the CO_2_PR intermediate state and catalyst. Taking 450 eV as the plane-wave cut-off energy during structural relaxation. The convergence criteria for geometric optimisation were set to (1) energy tolerance of 1.0 × 10^−5^ eV, (2) maximum force tolerance of −0.02 eV/Å, and (3) Monkhorst−Pack k-point sampling: 3 × 3 × 1. The *E*_form_ was defined as

*E*_form_ = *E*_doped_ + ∑*μ*_B_ − *E*_pure_ − ∑*μ*_A_ (1)

here *E*_pure_ and *E*_doped_ represent the energies of the catalysts before and after doping, respectively. The lowest energy doping structure is used as an example, where *E*_pure_ and *E*_doped_ are the energies of CN and KBCN, respectively, and *µ*_A_ and *µ*_B_ are the chemical potentials of the substituted atoms, the former for the C atom and the latter for the B and K atoms. The formation energy reflects the ease of doping, with lower energies representing easier catalyst production. The change in Δ*G* [54] was defined as

Δ*G* = Δ*E* + Δ*E*_zpe_ − TΔ*S* + Δ*G*_U_ + Δ*G*_pH_ (2)

where Δ*E* is the change in reaction energy obtained from DFT total electron energies, Δ*E*_ZPE_ represents the change in the zero-energy correction value, T is the room temperature, and Δ*S* is the change in entropy. Δ*G*_U_ = −n*eU*, where n is the number of transferred electrons, *e* is the elementary charge, and *U* is the electrode potential. Δ*G*_pH_ is the correction of the hydrogen proton free energy by the concentration, Δ*G*_pH_ = *k*_B_T × ln10 × pH, where *k*_B_ is the Boltzmann constant, and pH is set to zero in acidic conditions. The zero-point energy and entropy of the substances involved in the CO_2_PR process were calculated from the vibrational frequencies. When the catalyst substrate is fixed, the vibrational modes of the adsorbed substances are calculated exactly (assuming that the vibrations of the substrate can be neglected). The adsorption energies (*E*_ads_) of CO_2_ and the intermediates and products on CN and KBCN were calculated according to the following equation. Spin-polarised calculations were performed for all models of neutral states and radical intermediates involved in the adsorption process. With the spin-polarised parameter turned on, the VASP programme distinguishes between spin-up and spin-down eigenstates and treats them separately. In addition, the programme handles spin degrees of freedom by solving the spin-dependent Kohn–Sham equation and obtains different exchange-correlation potentials depending on the difference in spin density, which generates spin splitting to correctly describe unpaired electrons behaviour [55].

*E*_ads_ = *E*_A−S_ − *E*_A_ − *E*_S_ (3)

where *E*_A−S_, *E*_A_, and *E*_S_ are the total electron energies of the adsorbate–substrate (A−S) complex, absorbate (A), and substrate (S), respectively.

*Φ* = *E*_vacuum_ − *E*_fermi_ (4)

*E*_VBM_ = −*Φ* + 0.5 *E*_g_ (5)

*E*_CBM_ = −*Φ* − 0.5 *E*_g_ (6)

*E*’_CBM/VBM_ = *E*_CBM/VBM_ + 4.5 (7)

The work function and the position of the band edge can be calculated according to Equations (4)–(7) [35], where *Φ* is the work function, *E*_vacuum_ is the vacuum energy level, *E*_fermi_ is the Fermi energy level, *E*_g_ is the band gap, and *E’*_CBM/VBM_ is the potential with respect to the NHE (pH = 7).

For the energy band and density of states of catalysts, PBE tends to underestimate the gap between VBM and CBM. Therefore, to obtain more accurate band gap values, we used the Heyd–Scuseria–Ernzerhof (HSE06) hybrid functional method with 25% HF exchange, which has been demonstrated in previous studies to accurately predict the band gap of catalysts [35,56,57]. Other electronic structural properties, such as charge differential density and Bader charge, were calculated via the PBE method.

## 4. Conclusions

In summary, this work represents a comprehensive study of CO_2_PR based on DFT calculations for CN and KBCN, in which the effects of B and K doping on the electronic properties and catalytic performance of CO_2_PR were investigated. The band gap of KBCN is smaller than that of CN, predicting a red shift in the adsorption spectrum of KBCN compared to pristine CN. The HOMO of KBCN is mainly located in the heptazine structural unit where the B atom is located, while the LUMO is mainly located in the other two heptazine units, which do not contain the B element. The results indicate that KBCN is beneficial as a photocatalyst compared to CN in promoting charge separation, inhibiting electron–hole recombination and extending the lifetime of the charge carriers, thus improving photocatalytic efficiency. Compared with CN, the work function of KBCN is smaller than that of CN in all three directions, indicating that it is easier for KBCN to transport electrons and exhibit high mobility. DFT calculations show that CO_2_ is finally reduced to CH_3_OH under the catalysis of KBCN, and its optimal reduction path is CO_2_ → *CO_2_ → *COOH → CO → *OCH → HCHO → *OCH_3_ → CH_3_OH, where the rate-determining step is *CO_2_ → *COOH with a Δ*G* of 0.861 eV. Compared to CN, B and K doping altered the rate-determining step and decreased ∆*G*, thereby enhancing the CO_2_ reduction via KBCN. The results of this study can provide ideas for the design of efficient photocatalysts and important information for the production of industrial primary products for photocatalytic CO_2_ reduction.

## Data Availability

The data presented in this study are available in Supplementary Materials.

## Supplementary Materials

The following supporting information can be downloaded at: https://www.mdpi.com/article/10.3390/molecules29225339/s1, Table S1. The bond length parameters of CN and KBCN; Table S2. Fermi energy levels, vacuum energy levels, and work functions for CN and KBCN in the a,b,c directions; Figure S1. (a) AIMD simulates the energy changes in CN and KBCN with time at different temperatures. (b) The structure of CN was simulated before and after 500 K. (c) The structure of CN was simulated before and after 500 K; Figure S2. Projected density of states of (a,b) CN and (c–f) KBCN; Figure S3. Side reaction pathways in the reduction in CO_2_ by (a) CN and (b) KBCN; Figure S4. Bader charge variation for CO_2_PR on (a) CN and (b) KBCN, where positive values represent the electrons gained and negative values represent the electrons lost; Figure S5. (a) Gibbs free energy diagrams of photocatalytic HER of CN and KBCN; The representative geometries of the stable points in the reduction process for HER: (b) CN, (c) KBCN; Animations S1–S4: Video of AIMD simulations of CN and KBCN catalysts at room and 500 K temperatures.
