# Supplementary material for: Potassium and Boron Co-Doping of g-C3N4 Tuned CO2 Reduction Mechanism for Enhanced Photocatalytic Performance: A First-Principles Investigation
Source: Molecules. 2024 Nov 13;29(22):5339. doi: 10.3390/molecules29225339 (PMC11596514; doi:10.3390/molecules29225339)
Supplement: Supplementary file 1 [file molecules-29-05339-s001.zip › molecules-3278517-supplementary/molecules-3278517-supplementary.pdf]

Potassium and Boron Co-Doping of g-C_3N_4 Tuned CO_2 Reduction Mechanism for Enhanced Photocatalytic Performance: A First-Principles Investigation

Gang Fu,[1,2] Wenqing Zhen,[2] Hongyi Wang,[2] Xin Zhou,[2] Li Yang,[1,*] and Jiaxu Zhang[1,2,*]

[1] Xinjiang Key Laboratory of Clean Conversion and High Value Utilization of Biomass Resources, School of Chemistry and Chemical Engineering, Yi li Normal University, Yining 835000, P. R. China

[2] State Key Laboratory of Urban Water Resource and Environment, MIIT Key Laboratory of Critical Materials Technology for New Energy Conversion and Storage, School of Chemistry and Chemical Engineering, Harbin Institute of Technology, Harbin 150001, P. R. China

*Author E-mail Address: yangli2014@hit.edu.cn and zhjx@hit.edu.cn

Table S1. The bond length parameters of CN and KBCN.

Species			CN			KBCN	
Researcher	Our Work	Su[a]	Ding[b]	Zhang[c]	Thomas[d]	Researcher	Our Work
Cell parameter/Å	a=b=7.35	a=b=7.15	a=b=7.13	a=b=7.13	a=b=7.30	Cell parameter/Å	a=b=7.38
Bond length/Å	PBE	PBE	PW91	PBE	Expt	Bond length/Å	PBE
N1-C1	1.477	1.47	1.469	1.47	——	N1-B	1.676
C1-N2	1.350	1.34	1.337	1.33	——	B-N2	1.445
N2-C2	1.358	1.33	1.332	1.33	——	N2-C2	1.309
C2-N3	1.417	1.39	1.394	1.39	——	C2-N3	1.449
N-interstitial	2.522	——	——	——	——	N-K	2.673
C1-N	1.349	——	1.336	——	——	C1-N	1.442

Note: The numbers 1, 2, 3, 4, 5, 6, 7 and 8 represent the N1, C1, N2, C2, N3, C, N and interstitial positions respectively, which can be replaced by heteroatoms. [a,d] The parameters are from ref *The Journal of Physical Chemistry C*. 2018, 122, 7712-7719. [b] The parameters are from ref *Phys Chem Chem Phys*. 2016, 18, 19217-19226. [c] The parameters are from ref *Molecules*. 2022, 27, 7611.

Table S2. Fermi energy levels, vacuum energy levels and work functions for CN and KBCN in the a,b,c directions.

Species	E_{vacuum}	E_{Fermi}	Φ_{CN}	E_{vacuum}	E_{Fermi}	Φ_{KBCN}	$\Delta\Phi$
a	1.398	–3.320	4.718	1.542	–3.004	4.546	0.172
b	1.397	–3.320	4.717	1.488	–3.004	4.492	0.225
c	1.545	–3.320	4.865	1.724	–3.004	4.728	0.137

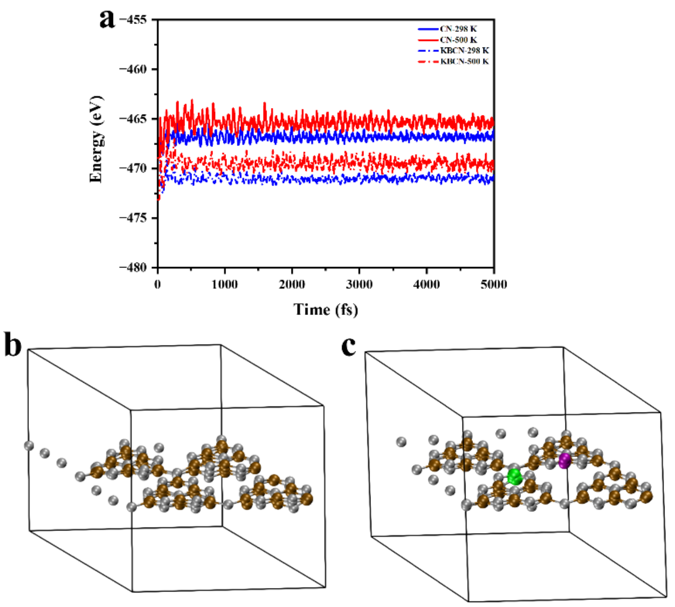

Fig. S1. (a) AIMD simulates the energy changes of CN and KBCN with time at different temperatures. (b) The structure of CN was simulated before and after 500K. (c) The structure of CN was simulated before and after 500K.

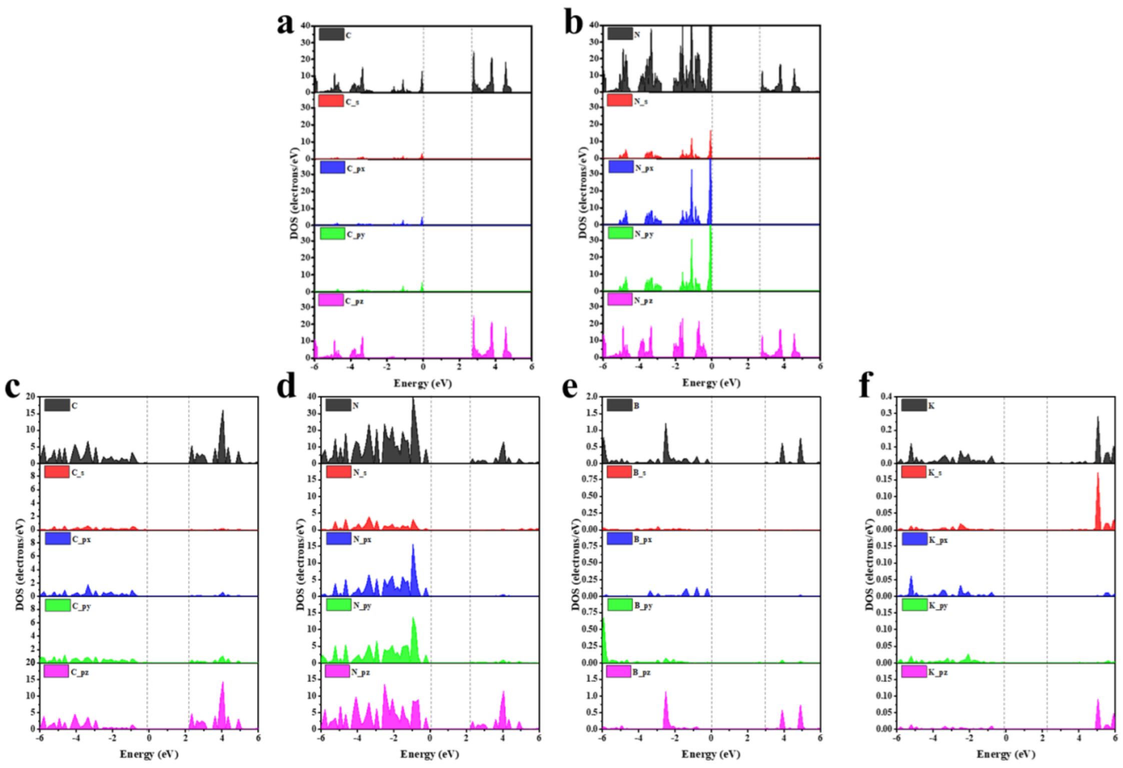

Fig. S2. Projected density of states of (a-b) CN and (c-f) KBCN.

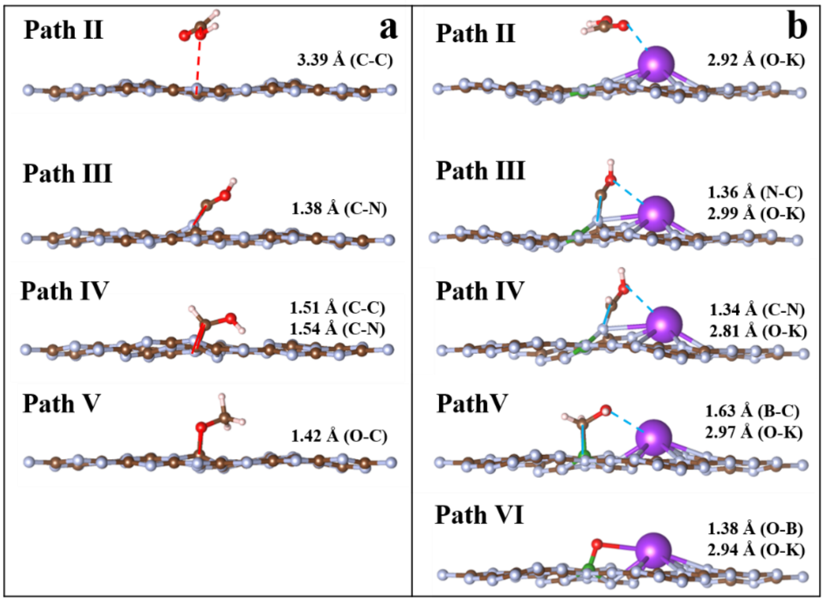

Fig. S3. Side reaction pathways in the reduction of CO_2 by (a) CN and (b) KBCN

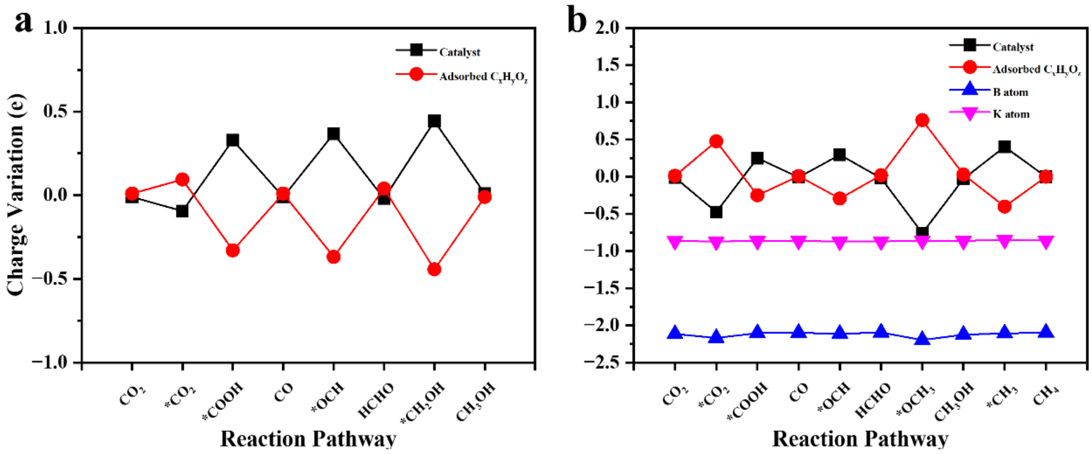

Fig. S4. Bader charge variation for CO_2PR on (a) CN and (b) KBCN, where positive values represent the electrons gained and negative values represent the electrons lost.

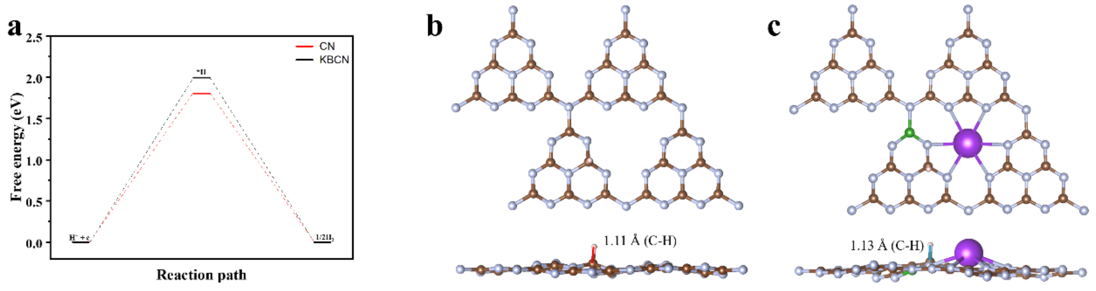

Fig. S5. (a) Gibbs free energy diagrams of photocatalytic HER of CN and KBCN; The reprsentative geometries of the stable points in the reduction process for HER: (b) CN, (c) KBCN.

Animation S1-S4 :

Animation S1: Video of AIMD simulations of CN catalysts at 298 K temperatures.

Animation S2: Video of AIMD simulations of CN catalysts at 500 K temperatures.

Animation S3: Video of AIMD simulations of KBCN catalysts at 298 K temperatures.

Animation S4: Video of AIMD simulations of KBCN catalysts at 500 K temperatures.
